# Associations of lipid metabolic parameters with cumulative live birth rate in PCOS patients undergoing IVF/ICSI and development of a nomogram-based predictive model

**DOI:** 10.3389/fendo.2026.1924206

**Published:** 2026-09-25

**Authors:** Xiaofeng Chen, Shumin Qiu, Lili Zheng, Jianqing Yang, Bangjing Huang, Beihong Zheng, Huiling Xu

**Affiliations:** 1 Center of Reproductive Medicine, Fujian Maternity and Child Health Hospital College of Clinical Medicine for Obstetrics & Gynecology and Pediatrics, Fujian Medical University, Fuzhou, Fujian, China; 2 Department of Maternal Health, Ningde Maternity and Child Health Hospital, Ningde, Fujian, China; 3 Department of Gynecology, Mindong Hospital Affiliated to Fujian Medical University, Ningde, Fujian, China

**Keywords:** assisted reproductive technology, cumulative live birth rate, dyslipidemia, nomogram, polycystic ovary syndrome

## Abstract

**Background:**

Polycystic ovary syndrome (PCOS) is a common endocrine disorder affecting reproductive-aged women, many of whom require *in vitro* fertilization (IVF) or intracytoplasmic sperm injection (ICSI) for infertility. Dyslipidemia frequently coexists with PCOS and may independently affect reproductive outcomes, yet evidence on its association with cumulative live birth rate (CLBR) remains limited. This study aimed to investigate the relationship between lipid metabolism parameters and CLBR in PCOS patients undergoing their first IVF/ICSI cycle, and to construct and validate a nomogram for individualized CLBR prediction.

**Methods:**

This retrospective analysis included 413 PCOS patients under 40 years old who underwent their first IVF/ICSI cycle between January 2022 and May 2024. The cohort was randomly split into a training set (n = 290) and a validation set (n = 123) at a 7:3 ratio. Lasso regression with 10-fold cross-validation was used for variable selection, followed by leave-one-variable-out sensitivity analysis. Multivariate logistic regression was then applied to build the prediction model. Internal validation employed bootstrap resampling with 1,000 iterations. Model performance was assessed by AUC, calibration curves, and decision curve analysis. A nomogram was constructed for clinical application.

**Result:**

Multivariate analysis identified high-quality embryo rate (OR = 1.025, 95% CI: 1.010–1.041, *P* = 0.001) and high-quality blastocyst rate (OR = 1.014, 95% CI: 1.002–1.026, *P* = 0.025) as independent protective factors. Elevated triglycerides (TG) (OR = 0.734, 95% CI: 0.555–0.969, *P* = 0.029) and higher BMI (OR = 0.915, 95% CI: 0.838–1.000, *P* = 0.049) were independent risk factors for reduced CLBR. The model achieved AUCs of 0.804 and 0.760 in the training and validation sets, respectively. Calibration was satisfactory (Brier score = 0.152), and DCA confirmed positive net benefit.

**Conclusion:**

Dyslipidemia, primarily elevated TG, is independently associated with lower CLBR in PCOS patients undergoing IVF/ICSI. Our nomogram incorporates TG, apolipoprotein A, BMI, trigger-day estradiol and progesterone, high-quality embryo rate and high-quality blastocyst rate. This tool may serve as a foundation for future external validation and exploratory risk stratification, though clinical application requires prospective multicenter studies.

## Introduction

In 2026, a global consensus formally renamed polycystic ovary syndrome (PCOS) to polyendocrine metabolic ovarian syndrome, recognizing the condition’s multisystem nature (1). Given the three-year transition period for this change and its continued widespread use in clinical practice (1, 2), we use the term PCOS throughout this manuscript. According to the 2003 Rotterdam criteria, PCOS affects 6% to 20% of reproductive-aged women worldwide (3, 4), making it one of the most common endocrine disorders in this population. Its clinical manifestations include oligomenorrhea, infertility, hyperandrogenism, obesity, insulin resistance, and dyslipidemia (5) reflecting the complex interplay between reproductive and metabolic dysfunction that underlies the condition.

For infertile patients with PCOS, *in vitro* fertilization (IVF) or intracytoplasmic sperm injection (ICSI) serves as a key assisted reproductive technology (ART). However, clinical observations reveal an interesting contrast. Compared to women whose infertility stems solely from tubal factors, those with PCOS who undergo IVF/ICSI tend to retrieve a larger number of oocytes. Yet this does not translate into a corresponding rise in clinical pregnancy rates (6). In addition, they face a markedly higher risk of early miscarriage (7). This suggest that beyond ovulation dysfunction, PCOS patients may have more profound reproductive defects. Therefore, it is essential to explore the various factors that influence ART outcomes in this population, with metabolic disturbances being a common concern.

Epidemiological data show that the overall prevalence of dyslipidemia among Chinese women with PCOS reaches as high as 41.3%,with low high-density lipoprotein (HDL) being the most frequent abnormality, accounting for about 57.4% of cases (8). Moreover, dyslipidemia is becoming increasingly common in PCOS patients, and this metabolic disorder can occur even in the absence of obesity. It may worsen other metabolic problems such as IR and hyperandrogenism, while also exerting long-term adverse effects on cardiovascular health (9). Recent attention has shifted toward the relationship between lipid metabolism disorders—such as elevated triglycerides (TG), total cholesterol (TC), low-density lipoprotein (LDL), and reduced HDL—and poor pregnancy outcomes after ART (10). Meanwhile, a high-fat diet has been shown to markedly increase both inflammatory responses and oxidative stress within follicles and oocytes (11). These changes trigger excessive inflammatory mediators, reactive oxygen species-driven apoptosis, and lipid peroxidation damage, ultimately impairing follicular and oocyte quality and thereby harming female reproductive function.

Currently, the impacts of obesity, IR, and dyslipidemia on pregnancy outcomes have been extensively discussed (12–14). However, studies specifically investigating the effect of dyslipidemia on IVF/ICSI outcomes in PCOS patients remain relatively scarce. Clinical management of PCOS patients often prioritizes weight control and insulin resistance (IR) intervention, while standardized management of lipid metabolism has not received sufficient attention. Furthermore, although predictive models for live birth following a single fresh embryo transfer in PCOS patients have been developed (15–17), these models primarily target live birth after a single transfer cycle. There is still a lack of systematic evaluation and relatively simple predictive tools for cumulative live birth rate (CLBR). Based on our previous work (16), this study aims to investigate the impact of abnormal lipid levels on the outcomes of PCOS patients undergoing their first IVF/ICSI cycles, and to develop and validate a multifactorial nomogram prediction model based on independent influencing factors to provide a quantitative tool for clinical decision-making.

## Materials and methods

### Study design and patients

This retrospective analysis included 413 PCOS patients under 40 years old who underwent their first IVF/ICSI cycle at our center between January 2022 and May 2024. Based on serum lipid profiles, patients were categorized into two groups: the dyslipidemia group (n=136) and the normal lipid group (n=277).

Inclusion criteria were as follows: (1) undergoing the first IVF/ICSI cycle; (2) receiving controlled ovarian hyperstimulation (COH) using either a follicular-phase long protocol or a GnRH antagonist (GnRH-A) protocol; (3) having a diagnosis of PCOS according to the Rotterdam criteria (18), defined as meeting at least two of the following three criteria: (1) oligo-ovulation or anovulation; (2) clinical and/or biochemical signs of hyperandrogenism; (3) polycystic ovaries on ultrasound, defined as the presence of ≥12 follicles measuring 2–9 mm in diameter in one or both ovaries and/or an ovarian volume ≥10 ml, with exclusion of other disorders causing hyperandrogenism and ovulatory dysfunction; (4) age<40 years.

Exclusion criteria were as follows: (1) history of chemotherapy, radiotherapy, or ovarian surgery; (2) presence of hydrosalpinx or uterine abnormalities; (3) history of adrenal disorders, thyroid disease, type 2 diabetes mellitus, Cushing’s syndrome, or other endocrine disorders; (4) history of autoimmune diseases or recurrent miscarriage; (5) preimplantation genetic testing; (6) *in vitro* maturation; (7) microsurgery sperm retrieval for azoospermia in the male partner or use of donor sperm.

### Sample size

This single-center retrospective study used Lasso regression for variable selection. The final model included eight predictors, with an outcome event rate of 75.8% (313/413) and 100 non-events, yielding an EPV of 12.5 (above the recommended ≥ 10 threshold). To confirm sample size adequacy, we applied the Riley method via the pmsampsize R package. Assuming an AUC of 0.804, eight predictors, and 75.8% event prevalence, the minimum required sample for a shrinkage factor ≥ 0.9 was 301 patients. Our cohort of 413 exceeded this, confirming sufficient statistical power.

### Protocols of IVF/ICSI

The included PCOS patients underwent COH using either a follicular phase long protocol or a GnRH-A protocol. ① Follicular-phase long protocol: A GnRH agonist was administered for downregulation on day 2–5 of the menstrual cycle. After successful downregulation, gonadotropin (Gn) was initiated individually based on age, BMI, and antral follicle count (AFC) to induce multiple follicular development. Follicular development and endometrial status were monitored regularly, and Gn was adjusted accordingly until the trigger day. ② GnRH-A protocol: Gn was initiated on day 2–5 of the menstrual cycle. GnRH-A was administered when the leading follicle reached 12–14 mm and serum estradiol (E2) ≥ 300 pg/mL, or luteinizing hormone(LH) >10 mIU/mL, or LH increased threefold from the baseline. When at least two follicles reached ≥18mm or three follicles reached ≥17mm, human chorionic gonadotropin (hCG) or GnRH agonist(only in GnRH-A protocol) was administered for final oocyte maturation. Transvaginal ultrasound-guided oocyte pick-up was performed 34–36 hours later.

Conventional IVF or ICSI was chosen based on semen parameters. Following oocyte retrieval, one or two day-3 embryos were selected for fresh transfer or frozen according to the patient’s condition. All remaining viable embryos were cultured to the blastocyst stage. Blastocysts with a score of 3CC or higher were considered usable; day-5 blastocysts were either transferred or frozen, while all day-6 blastocysts were frozen. Cleavage-stage embryo was evaluated according to the Istanbul Consensus, with embryos of ≥6 cells and grade II or higher classified as high-quality embryos (19). Blastocysts were scored according to the Gardner system, with blastocysts graded 3BB or higher considered high-quality (20).

Patients scheduled for fresh transfer received luteal phase support from the day of oocyte retrieval until 10–14 days after transfer. Fresh cycle embryo transfer was canceled, and all embryos were frozen in case of moderate or severe ovarian hyperstimulation syndrome (OHSS) (defined according to the American Society for Reproductive Medicine guidelines (21), a high risk of OHSS (defined as trigger-day E2 > 8000 pg/mL or > 25 oocytes retrieved, or 15–20 oocytes retrieved with E2 ≥ 5000 pg/mL accompanied by significant abdominal distension and ultrasound findings of markedly enlarged ovaries and ascites), high progesterone levels on the trigger day, or conditions such as vaginitis. Individuals who did not achieve a live birth in the fresh cycle but had frozen embryos were followed up for at least one year. These patients returned for frozen-thawed embryo transfer on days 2–5 of their second menstrual cycle following oocyte retrieval. Endometrial preparation was performed using either a hormone replacement cycle or an ovulation induction cycle.

### Pregnancy diagnosis

Serum β-hCG levels were measured 10–14 days after embryo transfer. A level above 25 mIU/mL was considered indicative of biochemical pregnancy. Ultrasound examination was performed 4–5 weeks after transfer, and the presence of an intrauterine gestational sac confirmed clinical pregnancy. Pregnancy loss before 28 weeks of gestation with a fetal weight of less than 1000 g was defined as a miscarriage. Live birth was defined as the delivery of a live infant at or after 28 weeks of gestation. Preterm birth referred to delivery between 28 and 37 weeks of gestation, while term birth referred to delivery at or after 37 weeks.

### Data collection and definitions

Blood samples were collected after at least 8 hours of fasting. Serum levels of TC, TG, HDL, LDL, apolipoprotein A (Apo-A), and apolipoprotein B (Apo-B) were measured using an automated biochemical analyzer (Heartcheck PA). Dyslipidemia was diagnosed according to the Chinese Guidelines for the Prevention and Treatment of Dyslipidemia in Adults (2016 Revised Edition) (20, 22), defined as having any one of the following: TC ≥ 6.20 mmol/L, TG ≥ 2.26 mmol/L, LDL ≥ 4.14 mmol/L, or HDL ≤ 1.04 mmol/L.

### Main outcome measures

Initial treatment cycle outcomes: first-transfer embryo implantation rate, first-transfer clinical pregnancy rate.

Cumulative treatment outcomes: clinical pregnancy rate, miscarriage rate, CLBR, preterm birth rate, and twin delivery rate.

Formula for calculated indicators


$$\text{BMI}=\text{Weight }\left(\text{kg}\right)/\text{Height }{\left(\text{m}\right)}^{2};$$

Homeostatic model assessment−insulin resistance (HOMA−IR)= fasting insulin (μIU/mL) × fasting glucose (mmol/L)/22.5;

High‐quality embryo rate =(number of high−quality day−3 embryos/number of MII oocytes) × 100%;

High-quality blastocyst rate = (number of high-quality blastocysts/number of day−3 embryos used for blastocyst culture) × 100% [Baseline comparisons used count-based embryo metrics to reflect absolute yield differences. For Lasso regression and the prediction model, percentages were adopted instead, to avoid multicollinearity with count variables (e.g., oocyte yield, MII count) and to eliminate confounding from individual variations in oocyte yield.]


OHSS incidence= (number of cycle with OHSS/number of COH cycles) × 100%

First − transfer embryo implantation rate = (number of gestational sacs after first transfer / total number of embryo transffered in the first transfer cycle, including the fresh cycle and the first frozen − thawed cycle after whole embryo freezing) × 100 %;

First-transfer clinical pregnancy rate = (number of cycles achieving clinical pregnancy in the first transfer / total number of the first transfer cycle , including the fresh cycle and the first frozen − thawed cycle after whole embryo freezing) × 100 %;


Clinical pregnancy rate= (number of cycles achieving clinical pregnancy / total number of transfer cycles) × 100%;

Miscarriage rate= (number of miscarriage cycles/number of clinical pregnancy cycles) × 100%;

CLBR= (number of patients achieving first live birth/total number of patients entering COH cycles) × 100%; [Live birth was defined as the delivery of a fetus with any vital signs, including heartbeat, respiration, or umbilical cord pulsation, regardless of gestational age or birth weight. Cases with gestational age<28 weeks and birth weight<1000 g were counted as live births if vital signs were present at delivery; otherwise, they were classified as miscarriages (including terminations due to fetal anomalies)]


Preterm birth rate= (number of preterm births/total number of deliveries) × 100%;

Twin delivery rate= (number of twin deliveries/total number of deliveries) × 100%.

### Statistical analysis

Statistical analyses were performed using SPSS software(version 26.0) and R software (version 4.5.1). Normally distributed continuous variables were presented as mean ± standard deviation (Mean ± SD), and comparisons between groups were performed using Student’s t-test. Non-normally distributed continuous variables were presented as median (Q1, Q3), and comparisons between groups were performed using the Mann-Whitney U test. Categorical variables are presented as frequency (percentage), and comparisons between groups were performed using the chi-square test or Fisher’s exact test.

Baseline characteristics and ovarian response parameters were compared between patients with and without live birth. Those with P< 0.2 or considered clinically relevant were entered into the Lasso regression. The 18 candidate variables included: age, BMI, HOMA-IR, duration of infertility, AMH, AFC, b-LH, Testosterone, Total Gn dose, HDL, TG, TC, LDL, Apo-A, E2 and P on the trigger day, High-quality embryo rate, High-quality blastocyst rate. Missing continuous data were imputed with the median; categorical data with the mode.

Lasso regression with 10-fold cross-validation (lambda.min criterion) selected 11 with non-zero coefficients from the initial set of 18 candidates. A subsequent leave-one-variable-out (LOVO) sensitivity analysis further refined the selection, excluding variables whose removal did not compromise the bootstrap-corrected AUC. This process retained eight predictors for inclusion in the multivariate logistic model. Internal validation was conducted via bootstrap resampling with 1,000 iterations to derive an optimism-corrected C-index. Model performance was assessed in both training and validation cohorts using ROC-derived AUC for discrimination, calibration plots combined with the Hosmer-Lemeshow test for calibration, and decision curve analysis for clinical net benefit. A nomogram was constructed from the final model to facilitate individualized CLBR prediction.

Multivariable logistic regression analysis was performed using SPSS to identify independent factors associated with CLBR. All modeling and validation procedures were conducted using R software, with the following packages: glmnet (Lasso regression), rms (logistic regression and nomogram), pROC (ROC curves and AUC), and rmda (decision curve analysis). A two-sided *P*< 0.05 was considered statistically significant.

## Results

This study included a total of 413 PCOS patients undergoing their first IVF/ICSI cycle using either a follicular phase long protocol or a GnRH-A protocol. Among these patients, 136 were in the dyslipidemia group and 277 in the normal lipid group. The occurrence of abnormalities in different lipid components within the dyslipidemia group is presented in **Figure 1**. Fresh embryo transfer was performed in 158 cycles, while frozen-thawed embryo transfer was performed in 461 cycles. In total, 201 transfer cycles were performed in the dyslipidemia group and 418 in the normal lipid group. In this study, 4 cases had a gestational age between 24 and 28 weeks with birth weight<1000 g. One case (twin pregnancy at 27^+1^ weeks) presented with vital signs at delivery and was counted as a live birth; the remaining 3 cases (24–27 weeks) underwent termination due to fetal anomalies with no vital signs at delivery and were classified as miscarriages.

**Figure 1:**
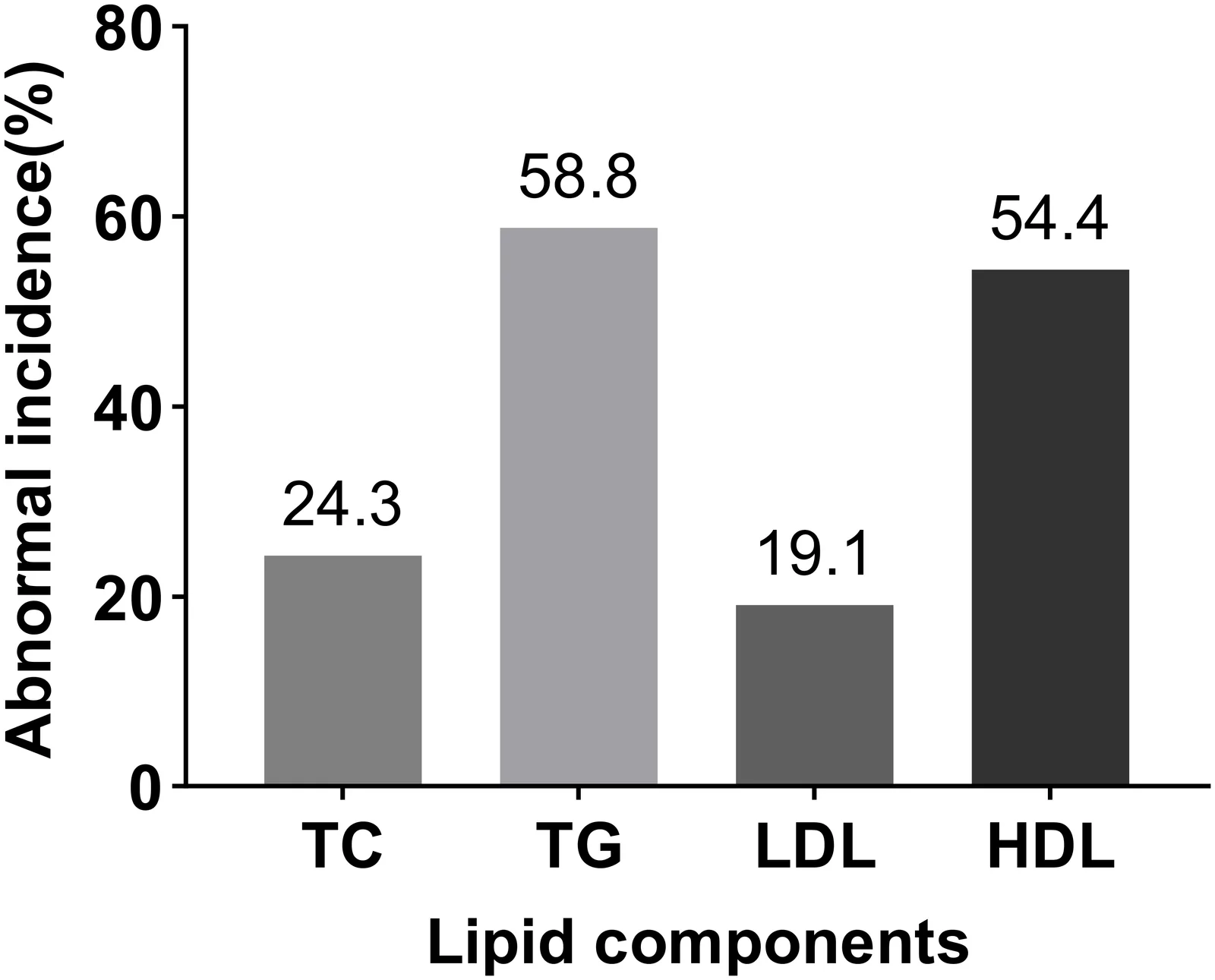
Incidence of abnormalities in different lipid components among PCOS patients with dyslipidemia. TC, total cholesterol; TG, triglycerides; HDL, high-density lipoprotein; LDL, low-density lipoprotein.

### Comparison of baseline characteristics between PCOS patients with and without dyslipidemia

Baseline characteristics of both groups are detailed in **Table 1**. The dyslipidemia group had a significantly higher BMI and HOMA-IR compared to the normal group. Basal LH, basal progesterone(P), and prolactin (PRL) levels were significantly lower in the dyslipidemia group. The intergroup differences in lipid profiles were as expected: the dyslipidemia group had significantly higher levels of TC, TG, LDL, and Apo-B, while HDL and Apo-A levels were significantly lower (all *P<* 0.01). There were no significant differences between the two groups in age, duration of infertility, type of infertility, AFC, basal follicle-stimulating hormone(FSH), basal E2, testosterone(T), or AMH levels (*P* > 0.05).

**Table 1:** Comparisons of baseline characteristics between PCOS patients with and without dyslipidemia.

Characteristics	Dyslipidemia group(*n* = 136)	Normal group(*n* = 277)	*z/t/χ²*	*P*
Age (years)	31.0 (29.0,33.5)	30.0 (28.0,32.0)	-1.729	0.084
BMI (kg/m^2^)	24.6 (22.0,27.3)	22.1 (20.3,24.7)	-5.925	**<0.001**
Duration of infertility (years)	3.0 (2.0,5.0)	3.0 (2.0,5.0)	-0.918	0.359
Infertility type (%)			1.023	0.312
Primary	63.2 (86/136)	68.2 (189/277)		
Secondary	36.8 (50/136)	31.8 (88/277)		
AFC (n)	27.0 (23.0,34.8)	27.0 (23.0,34.0)	-0.159	0.874
b FSH (IU/L)	5.2 (4.3,6.2)	5.3 (4.6,6.3)	-1.537	0.124
b LH (IU/L)	4.3 (2.9,6.5)	5.0 (3.4,7.2)	-2.194	**0.028**
PRL (ng/ml)	12.3 (9.1,18.5)	14.2 (10.4,21.1)	-2.993	**0.003**
b E2 (pg/ml)	30.0 (22.3,36.0)	30.0 (22.0,42.5)	-0.283	0.777
b P (ng/ml)	0.19 (0.1,0.3)	0.23 (0.1,0.3)	-3.434	**0.001**
T (pg/ml)	0.4 (0.3,0.5)	0.4 (0.3,0.5)	-0.600	0.549
AMH (ng/ml)	8.8 (6.1,13.2)	9.8 (7.0,13.2)	-0.669	0.504
HOMA-IR	2.73 (1.81,4.01)	1.84 (1.34,2.55)	-6.281	**<0.001**
TC (mmol/L)	5.20 (4.4,6.2)	4.81 (4.3,5.3)	-4.573	**<0.001**
TG (mmol/L)	2.44 (1.6,3.0)	1.08 (0.8,1.5)	-12.697	**<0.001**
LDL (mmol/L)	3.27 ± 0.83	2.83 ± 0.55	-5.614	**<0.001**
HDL (mmol/L)	1.08 (0.9,1.3)	1.41 (1.2,1.6)	-8.784	**<0.001**
Apo-A (g/l)	1.07 (1.0,1.2)	1.19 (1.1,1.3)	-4.619	**<0.001**
Apo-B (g/l)	0.96 (0.8,1.1)	0.80 (0.7,0.9)	-7.404	**<0.001**

Values are median (D1, D3) for continuous variables, or % (n/N) for categorical variables. BMI, body mass index; AFC, antral follicle count; b FSH, basal follicle-stimulating hormone; b LH, basal luteinizing hormone; b E2, basal estradiol; b P, basal progesterone; PRL, prolactin; T, serum total testosterone; AMH, anti-Müllerian hormone; HOMA-IR, the homeostasis model assessment of insulin resistance index; Apo-A, apolipoprotein A; Apo-B, apolipoprotein B. Bold values indicate statistical significance (P < 0.05).

### Comparison of ovarian response and pregnancy outcomes between PCOS patients with and without dyslipidemia

The comparisons of ovarian response and pregnancy outcomes between the two groups are detailed in **Table 2**. Regarding ovarian response, the dyslipidemia group required a significantly higher total Gn doses, while the FSH levels, E2 levels, and endometrial thickness on the trigger day, as well as the numbers of retrieved oocytes, MII oocytes, high-quality embryos, high-quality blastocysts, and frozen embryos were all significantly lower than those in the normal group (all *P<* 0.05). There were no significant differences between the two groups in the number of Gn stimulation days, LH and P levels on the trigger day, stimulation protocol, or insemination method (*P* > 0.05). Regarding pregnancy outcomes, the dyslipidemia group had a significantly lower CLBR and first-transfer embryo implantation rate, while the preterm birth rate was significantly higher (*P<* 0.05). The first-transfer clinical pregnancy rate and overall clinical pregnancy rate in the dyslipidemia group were numerically lower than those in the normal group (57.4% vs. 66.1%; 56.7% vs. 63.4%), but the differences were not statistically significant (*P* > 0.05). The incidence of OHSS, miscarriage rate, and twin delivery rate were similar between the two groups (*P* > 0.05).

**Table 2:** Comparison of ovarian hyperstimulation and pregnancy outcomes between PCOS patients with and without dyslipidemia.

Variable	Dyslipidemia group(*n* = 136)	Normal group(*n* = 277)	*z/χ²*	*P*
COH protocols (%)			1.422	0.233
Follicular-phase long protocol	38.2 (52/136)	44.4 (123/277)		
GnRH-A protocol	61.8 (84/136)	55.6 (154/277)		
Insemination method (%)			0.641	0.423
IVF	69.9 (95/136)	74.7 (207/277)		
ICSI	30.2 (41/136)	25.3 (70/277)		
Total Gn dose (IU)	2100 (1625,2750)	1875 (1500,2475)	-2.492	**0.013**
Gn stimulation (days)	12 (10.0,13.0)	11 (10.0,13.0)	-1.385	0.166
FSH on the trigger day (IU/L)	9.03 (7.3,10.9)	9.36 (7.9,11.8)	-2.494	**0.013**
LH on the trigger day (IU/L)	1.31 (0.8,2.1)	1.28 (0.8,2.0)	-0.421	0.674
E2 on the trigger day (pg/ml)	4037.0 (2500.8,5683.3)	4829.0 (3228.0,7111.5)	-3.225	**0.001**
P on the trigger day (ng/ml)	0.79 (0.5,1.4)	0.94 (0.6,1.4)	-1.458	0.145
Endometrial thickness on the trigger day (mm)	11.0 (9.8,12.5)	11.7 (10.0,13.0)	-2.187	**0.029**
NO. of retrieved oocytes	17 (12,24)	20 (14,26)	-2.776	**0.006**
NO. of MII-stage oocytes	15 (9,21)	17 (12,22)	-2.362	**0.018**
NO. of high-quality embryos	8 (4,12)	9 (6,14)	-2.643	**0.008**
NO. of high-quality blastocysts	2 (0,5)	4 (1,6)	-2.868	**0.004**
NO. of frozen embryos	5 (2,8)	6 (3,9)	-2.462	**0.014**
OHSS incidence (%)	7.35 (10/136)	9.75 (27/277)	0.641	0.423
First-transfer embryo implantation rate (%)	44.0 (84/191)	55.7 (194/348)	6.838	**0.009**
First-transfer clinical pregnancy rate (%)	57.4 (78/136)	66.1 (183/277)	2.977	0.084
Clinical pregnancy rate (%)	56.7 (114/201)	63.4 (265/418)	2.552	0.110
Miscarriage rate (%)	19.3 (22/114)	15.1 (40/265)	1.030	0.310
CLBR (%)	65.4 (89/136)	80.9 (224/277)	11.827	**0.001**
Preterm birth rate (%)	27.0 (24/89)	16.5 (37/224)	4.432	**0.035**
Twin delivery rate (%)	10.1 (9/89)	8.9 (20/224)	0.106	0.745

Values are median (D1, D3) for continuous variables, or % (n/N) for categorical variables. IVF, *in vitro* fertilization; ICSI, intracytoplasmic sperm injection; Gn, gonadotropin; hCG, human chorionic gonadotropin; FSH, follicle-stimulating hormone; LH, luteinizing hormone; E2, estradiol; P, progesterone; OHSS, ovarian hyperstimulation syndrome; CLBR, cumulative live birth rate. Bold values indicate statistical significance (P < 0.05).

### Stratified analysis of the impact of dyslipidemia by BMI

To clarify whether the impact of dyslipidemia is independent of weight status, patients were stratified into overweight/obese (BMI ≥ 25 kg/m²) and normal weight (BMI< 25 kg/m²) groups according to World Health Organization criteria for stratified analysis. As shown in **Table 3**, within the overweight/obese group, patients with dyslipidemia had significantly lower numbers of retrieved oocytes, MII oocytes, high-quality embryos, and high-quality blastocysts compared to those with normal lipids (*P<* 0.05). In the normal weight group, patients with dyslipidemia demonstrated significantly lower FSH, E2, and P levels on the trigger day, as well as a thinner endometrium, compared to those with normal lipids (*P<* 0.05). Regarding pregnancy outcomes, within the overweight/obese group, patients with dyslipidemia had significantly lower first-transfer embryo implantation rates, clinical pregnancy rates, and CLBR(*P<* 0.05). The first-transfer clinical pregnancy rate also showed a decreasing trend (47.6% vs. 59.6%), but the difference was not statistically significant. In the normal weight group, except for the preterm birth rate, there were no significant differences in other pregnancy outcome indicators.

**Table 3:** Comparison of ovarian hyperstimulation and pregnancy outcomes between PCOS patients in different BMI groups with and without dyslipidemia.

Variables	Overweight/obese group		*P*	Normal weight group		*P*
	Dyslipidemia group(n=65)	Normal group(n=62)		Dyslipidemia group(n=71)	Normal group (n=215)	
Total Gn dose (IU)	2437.5 (1950.0,2925.0)	2362.5 (1875.0,3150.0)	0.973	1775.0 (1475.0,2375.0)	1725.0 (1450.0,2275.0)	0.532
Gn stimulation (days)	12 (10.5,13.0)	12 (10.0,14.0)	0.813	11 (10.0,13.0)	11 (10.0,13.0)	0.622
FSH on the trigger day (IU/L)	9.32 (7.3,11.1)	9.15 (7.4,11.4)	0.772	8.96 (7.4,10.8)	9.43 (8.3,11.9)	**0.012**
LH on the trigger day (IU/L)	1.53 (0.8,2.9)	1.40 (0.8,2.5)	0.550	0.99 (0.7,1.6)	1.28 (0.8,1.9)	0.062
E2 on the trigger day (pg/ml)	3715.0 (2240.0,4932.5)	4328.5 (2449.8,5933.5)	0.337	4420.0 (2664.0,6630.0)	5022.0 (3632.0,7263.0)	**0.045**
P on the trigger day (ng/ml)	0.80 (0.6,1.6)	0.89 (0.5,1.4)	0.808	0.71 (0.5,1.3)	0.97 (0.6,1.4)	**0.048**
Endometrial thickness on the trigger day (mm)	11.60 ± 2.09	12.02 ± 2.04	0.255	10.90 ± 1.74	11.49 ± 1.91	**0.022**
NO. of retrieved oocytes	16 (11,21.5)	18.5 (14,26)	**0.019**	19.0 (13.0,27.0)	20.0 (15.0,26.0)	0.306
NO. of MII-stage oocytes	13 (8,18)	16.5 (12,22)	**0.016**	17 (10,23)	17 (12,23)	0.789
NO. of high-quality embryos	7.0 (3.0,10.5)	10.0 (5.0,13.0)	**0.044**	8.0 (6.0,12.0)	9.0 (6.0,14.0)	0.229
NO. of high-quality blastocysts	2.0 (0.0,5.0)	4.0 (1.0,6.0)	**0.030**	3.0 (1.0,5.0)	3.0 (1.0,6.0)	0.120
NO. of frozen embryos	5.0 (1.5,8.0)	6.0 (3.75,9.0)	0.080	6.0 (3.0,8.0)	6.0 (3.0,10.0)	0.131
OHSS Incidence (%)	6.2 (4/65)	4.8 (3/62)	1.000	8.59 (6/71)	11.2 (24/215)	0.518
First-transfer embryo implantation rate (%)	35.5 (33/93)	54.8 (46/84)	**0.010**	52.0 (51/98)	56.0 (149/266)	0.499
First-transfer clinical pregnancy rate (%)	47.6 (31/65)	59.6 (37/62)	0.176	66.1 (47/71)	67.9 (146/215)	0.79
Clinical pregnancy rate (%)	47.5 (47/99)	61.9 (60/97)	**0.043**	65.7 (67/102)	63.9 (205/321)	0.738
Miscarriage rate (%)	21.3 (10/47)	16.7 (10/60)	0.544	17.9 (12/67)	14.6 (30/205)	0.519
CLBR (%)	53.9 (35/65)	79.0 (49/62)	**0.003**	76.1 (54/71)	81.4 (175/21)	0.329
Preterm birth rate (%)	22.99 (8/35)	30.6 (15/49)	0.432	29.6 (16/54)	12.6 (22/175)	**0.003**
Twin delivery rate (%)	5.7 (2/35)	18.4 (9/49)	0.09	13.0 (7/54)	6.3 (11/175)	0.192

Values are median (D1, D3) for continuous variables, or % (n/N) for categorical variables. Bold values indicate statistical significance (P < 0.05).

### Stratified analysis of the impact of dyslipidemia by HOMA-IR

Patients were divided into an IR group (HOMA-IR ≥ 2.69) and a non-IR group (HOMA-IR<2.69) based on HOMA-IR. As shown in **Table 4**, within the IR group, patients with dyslipidemia had significantly lower numbers of high-quality embryos and blastocysts compared to those with normal lipids (*P*< 0.05). In the non-IR group, patients with dyslipidemia had significantly lower FSH and E2 levels on the trigger day compared to those with normal lipids (*P<* 0.05). Regarding pregnancy outcomes, within the IR group, patients with dyslipidemia showed a trend toward lower CLBR (61.4% vs. 75.8%), but the difference was not statistically significant *(P* = 0.077). In contrast, within the non-IR group, patients with dyslipidemia had a significantly lower CLBR than those with normal lipids (69.7% vs. 82.3%, *P* = 0.027) and their preterm birth rate was significantly higher (28.3% vs. 15.3%, *P<* 0.05). There were no significant differences between the two groups in OHSS incidence, first-transfer embryo implantation rate, first-transfer clinical pregnancy rate, miscarriage rate, clinical pregnancy rate, or twin delivery rate (all *P* > 0.05).

**Table 4:** Comparison of ovarian hyperstimulation and pregnancy outcomes between PCOS patients in different HOMA-IR groups with and without dyslipidemia.

Variables	IR group		*P*	Non-IR group		*P*
	Dyslipidemia group(n=70)	Normal group (n=62)		Dyslipidemia group (n=66)	Normal group (n=215)	
Total Gn dose (IU)	2237.5 (1650.0,2931.3)	2025.0 (1643.8,2925.0)	0.333	1950.0 (1562.5,2550.0)	1825.0 (1500.0,2400.0)	0.165
Gn stimulation (days)	12 (10.8,13.0)	11.0 (10.0,13.0)	0.103	11.0 (10.0,13.0)	11.0 (10.0,13.0)	0.872
FSH on the trigger day (IU/L)	9.07 (7.2,10.8)	9.02 (7.8,10.6)	0.626	8.99 (7.4,11.0)	9.47 (7.9,12.1)	**0.045**
LH on the trigger day (IU/L)	1.42 (0.9,2.8)	1.49 (0.8,2.7)	0.648	1.00 (0.7,1.8)	1.26 (0.8,1.9)	0.059
E2 on the trigger day (pg/ml)	3954.0 (2595.5,5418.5)	4478.5 (2618.3,6106.0)	0.441	4354.0 (2190.3,6541.8)	4954.0 (3471.0,7307.0)	**0.019**
P on the trigger day (ng/ml)	0.80 (0.6,1.6)	0.93 (0.5,1.3)	0.962	0.71 (0.4,1.3)	0.97 (0.6,1.4)	0.064
Endometrial thickness on the trigger day (mm)	11.53 ± 2.08	11.57 ± 1.94	0.907	11.60 ± 2.09	12.02 ± 2.04	0.255
NO. of retrieved oocytes	17.0 (12.0,24.0)	20.5 (14.0,26.0)	0.056	17.0 (12.0,25.0)	20.0 (14.0,26.0)	0.104
NO. of MII-stage oocytes	14.0 (10.0,20.0)	15.5 (11.0,22.0)	0.189	15.0 (9.0,21.0)	17.0 (12.0,23.0)	0.121
NO. of high-quality embryos	7.0 (4.0,11.3)	9.0 (6.0,13.3)	**0.044**	8.0 (4.0,12.0)	9.0 (6.0,14.0)	0.161
NO. of high-quality blastocysts	2.0 (0,5.0)	3.0 (1.0,6.0)	**0.026**	3.0 (0,6.0)	4.0 (1.0,6.0)	0.126
NO. of frozen embryos	5.0 (2.0,8.0)	6.0 (3.0,8.0)	0.216	5.0 (2.0,8.0)	6.0 (3.0,10.0)	0.075
OHSS Incidence (%)	7.1 (5/70)	4.8 (3/62)	0.851	7.6 (5/66)	11.2 (24/215)	0.402
First-transfer embryo implantation rate (%)	46.1 (47/102)	56.4 (44/78)	0.169	44.9 (40/89)	55.6 (150/270)	0.082
First-transfer clinical pregnancy rate (%)	55.7 (39/70)	61.2 (38/62)	0.517	59.0 (39/66)	67.4 (145/215)	0.212
Clinical pregnancy rate (%)	52.8 (57/108)	60.4 (55/91)	0.278	61.3 (57/93)	64.2 (210/327)	0.604
Miscarriage rate (%)	21.1 (12/57)	12.7 (7/55)	0.241	17.5 (10/57)	15.7 (33/210)	0.739
CLBR (%)	61.4 (43/70)	75.8 (47/62)	0.077	69.7 (46/66)	82.3 (177/215)	**0.027**
Preterm birth rate (%)	25.6 (11/43)	21.3 (10/47)	0.630	28.3 (13/46)	15.3 (27/177)	**0.041**
Twin delivery rate (%)	9.3 (4/43)	6.4 (3/47)	0.902	10.9 (5/46)	9.6 (17/177)	1.000

Values are median (D1, D3) for continuous variables, or % (n/N) for categorical variables. Bold values indicate statistical significance (P < 0.05).

### Baseline characteristics and ovarian response of PCOS patients by live birth outcome

Patients were divided into two groups according to whether they achieved a live birth: the live-birth group (n = 313) and the non-live-birth group (n = 100). **Tables 5A, B** summarizes the baseline characteristics and ovarian response comparisons between the two groups. Regarding general and endocrine characteristics, the live-birth group had a significantly lower BMI (22.6 vs. 24.0 kg/m², *P* = 0.001), whereas their levels of bLH, T, and AMH were significantly higher. No significant differences were observed between the two groups in age, infertility duration, infertility type, AFC, bFSH, PRL, bE2, or bP (all *P* > 0.05). With respect to metabolic parameters, the live-birth group showed lower HOMA-IR and TG levels, along with higher HDL and Apo-A levels, compared to the non-live-birth group (all *P*< 0.05). TC, LDL, and Apo-B did not differ significantly between the two groups (all *P* > 0.05). As for ovarian stimulation and embryological outcomes, the live-birth group had significantly higher values for trigger-day E_2_ and P, oocyte yield, MII oocyte count, high-quality embryo count, high-quality blastocyst count, and frozen embryo count (all *P*< 0.01). No significant intergroup differences were seen for stimulation protocol, fertilization method, total Gn dose, Gn duration, trigger-day FSH, trigger-day LH, or endometrial thickness on the trigger day (all *P* > 0.05).

**Table 5A:** Comparisons of baseline characteristics between PCOS patients with and without live birth.

Variables	Non-live birth group(*n* = 100)	Live birth group(*n* = 313)	*z/X^2^*	*P*
Age (years)	30.0 (27.0,33.0)	31.0 (28.0,33.0)	-0.428	0.669
BMI (kg/m^2^)	24.0 (21.0,27.3)	22.6 (20.6,25.3)	-3.397	**0.001**
Duration of infertility (years)	4.0 (2.0,5.0)	3.0 (2.0,5.0)	-1.644	0.100
Infertility type (%)			0.346	0.557
Primary	69 (69/100)	65.8 (206/313)		
Secondary	31 (31/100)	34.2 (107/313)		
AFC (n)	26.0 (22.3,31.0)	27.0 (23.0,35.0)	-1.750	0.080
b FSH (U/L)	5.37 (4.7,6.4)	5.27 (4.5,6.2)	-0.555	0.579
b LH (U/L)	3.79 (2.6,6.3)	4.95 (3.4,7.2)	-2.717	**0.007**
PRL (ng/ml)	13.25 (10.4,18.9)	13.70(10.0,20.8)	-0.918	0.359
b E2 (pg/ml)	29.000 (23.3,39.8)	31.000 (21.0,40.5)	-0.041	0.967
b P (ng/ml)	0.200 (0.1,0.3)	0.200 (0.1,0.3)	-0.710	0.478
T (pg/ml)	0.375 (0.3,0.5)	0.400 (0.3,0.5)	-2.127	**0.033**
AMH (ng/ml)	8.140 (5.3,12.1)	9.880 (7.0,13.5)	-2.993	**0.003**
HOMA-IR	2.300 (1.7,3.5)	2.020 (1.4,2.9)	-2.560	**0.01**
TC (mmol/L)	4.835 (4.2,5.5)	4.890 (4.4,5.4)	-0.643	0.520
TG (mmol/L)	1.520 (1.1,2.7)	1.220 (0.9,1.8)	-4.085	**<0.001**
LDL (mmol/L)	3.000 (2.5,3.4)	2.860 (2.5,3.4)	-0.764	0.445
HDL (mmol/L)	1.180 (1.0,1.4)	1.340 (1.1,1.6)	-4.228	**<0.001**
Apo-A (g/L)	1.110 (1.0,1.2)	1.170 (1.1,1.3)	-3.252	**0.001**
Apo-B (g/L)	0.850 (0.7,1.0)	0.805 (0.7,1.0)	-0.742	0.458

Values are median (D1, D3) for continuous variables, or % (n/N) for categorical variables. Bold values indicate statistical significance (P < 0.05).

**Table 5B:** Comparisons of ovarian hyperstimulation between PCOS patients with and without live birth.

Variables	Non-live birth group(*n* = 100)	Live birth group(*n* = 313)	*z/X^2^*	*P*
COH protocols (%)			0.021	0.884
Follicular-phase long protocol	43.0(43/100)	42.2(132/313)		
GnRH-A protocol	57.0(57/100)	57.8(181/313)		
Insemination method (%)			0.001	0.974
IVF	73.0(73/100)	73.2(229/313)		
ICSI	27.0(27/100)	26.8(84/313)		
Total Gn dose (IU)	2112.5(1575.0,2768.8)	1875.0(1525.0,2475.0)	-1.641	0.101
Gn stimulation(days)	11.0(10.0,13.0)	11.0(10.0,13.0)	-0.166	0.868
FSH on the trigger day (IU/L)	9.54(7.6,11.8)	9.26(7.8,11.2)	-0.723	0.470
LH on the trigger day (IU/L)	1.34(0.8,2.3)	1.28(0.8,2.0)	-0.268	0.788
E2 on the trigger day (pg/ml)	3515.0(2223.5,4837.3)	4892.0(3445.5,7188.0)	-5.266	**<0.001**
P on the trigger day (ng/ml)	0.66(0.4,1.1)	0.98(0.6,1.4)	-4.117	**<0.001**
Endometrial thickness on the trigger day (mm)	11.0(10.0,12.7)	11.6(10.0,13.0)	-1.126	0.260
NO. of retrieved oocytes	14.0(10.0,20.0)	21.0(15.0,26.0)	-5.464	**<0.001**
NO. of MII-stage oocytes	13.0(7.3,17.0)	18.0(12.0,23.0)	-6.190	**<0.001**
NO. of high-quality embryos	5.0(3.0,8.0)	10.0(7.0,14.0)	-8.480	**<0.001**
NO. of high-quality blastocysts	1.0(0.0,3.0)	4.0(2.0,7.0)	-8.096	**<0.001**
NO. of frozen embryos	2.0(0.0,5.8)	7.0(4.0,10.0)	-8.442	**<0.001**

Values are median (D1, D3) for continuous variables, or % (n/N) for categorical variables. Bold values indicate statistical significance (P < 0.05).

### Lasso regression for variable selection

Eighteen candidate variables—those with *P*< 0.2 in univariate analysis or with recognized clinical relevance—were entered into the Lasso regression. The optimal penalty parameter λ was determined via 10-fold cross-validation using the lambda.min criterion (λ = 0.0224). This procedure yielded 11 variables with non-zero coefficients: age, BMI, AMH, APO-A, TG, HDL, E2, and P levels on the trigger day, high-quality embryo rate, and high-quality blastocyst rate. The Lasso path diagram and cross-validation error plot are presented in **Figures 2A, B**, while the coefficient distribution for each variable is shown in **Figure 2C**.

**Figure 2:**
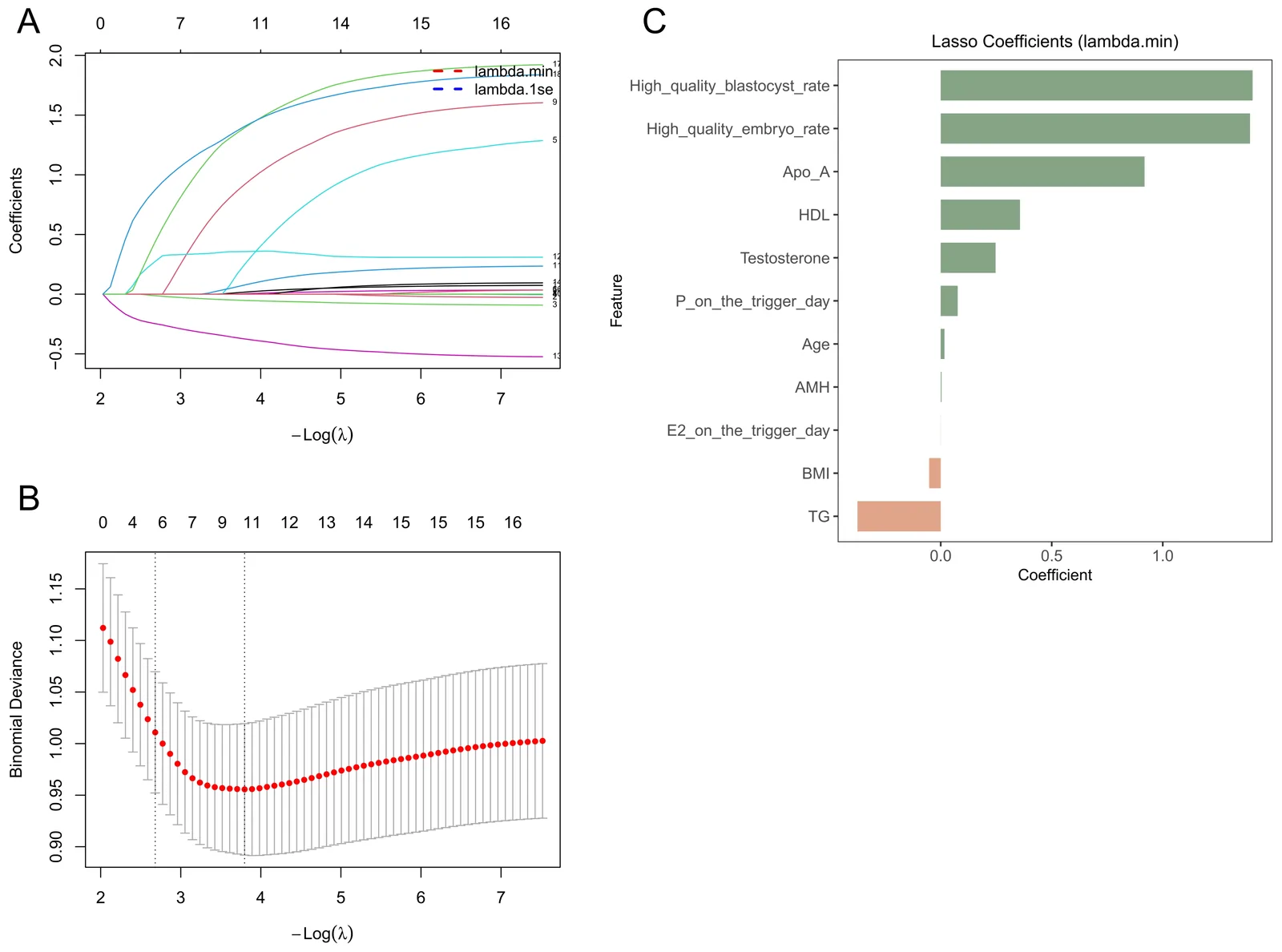
Lasso regression for variable selection. **(A)** Lasso regression path. The x-axis is shown as −Log(λ) (λ, penalty parameter), and the y-axis represents regression coefficients. The optimal penalty parameters selected by 10-fold cross-validation were λ.min = 0.0224 [corresponding to −Log(λ) = 3.80] and λ.1se = 0.0685 [corresponding to −Log(λ) = 2.68]. The final model was selected using λ.min (minimum error criterion) to retain more potentially relevant predictors. **(B)** Cross-validation deviance plot. The y-axis shows binomial deviance as a measure of model fit error, and the x-axis is −Log(λ). **(C)** Lasso coefficient plot. Only predictors with non-zero coefficients in the final model are displayed. Positive coefficients indicate a positive association with cumulative live birth (CLBR), whereas negative coefficients indicate a negative association.

### Model refinement via leave-one-variable-out analysis

To further optimize the model and enhance its clinical practicality, we performed a LOVO analysis to evaluate each variable’s individual contribution to model performance (**Supplementary Figure 1**). Removal of age, T, or HDL raised the validation-set AUC to 0.756, 0.752, and 0.748, respectively. These three variables were therefore excluded from the final model.

The streamlined model, comprising eight variables, achieved a raw AUC of 0.760 (95% CI: 0.653–0.867) in the validation set, with a bootstrap-corrected AUC of 0.775 and a Brier score of 0.152. By comparison, the full 11-variable Lasso model yielded corresponding values of 0.748 (95% CI: 0.639–0.857), 0.770, and 0.156 (**Table 6**).

**Table 6:** Validation performance comparison between the full lasso model and the final parsimonious model.

Model	Number of predictors	Validation AUC (95%)	Bootstra-corrected AUC	Brier score
Full Lasso model	11	0.748 (0.639-0.857)	0.770	0.156
Final parsimonious model	8	0.760 (0.653-0.867)	0.775	0.152

The final parsimonious model was derived from the full Lasso model by removing Age, Testosterone, and HDL based on leave-one-variable-out (LOVO) sensitivity analysis. AUC, area under the curve; CI, confidence interval.

### Multivariate logistic regression analysis

The eight variables retained after Lasso screening and LOVO refinement were entered into a multivariate logistic regression model. Results showed that high-quality embryo rate (OR = 1.025, 95% CI: 1.010–1.041, *P* = 0.001), high-quality blastocyst rate (OR = 1.014, 95% CI: 1.002–1.026, *P* = 0.025), TG (OR = 0.734, 95% CI: 0.555–0.969, *P* = 0.029), and BMI (OR = 0.915, 95% CI: 0.838–1.000, *P* = 0.049) were independently associated with CLBR (**Table 7**).

**Table 7:** Multivariable logistic regression analysis of factors associated with CLBR.

Variable	OR (95% CI)	*P* value
BMI	0.915 (0.838-1.000)	**0.049**
AMH	1.034 (0.971-1.102)	0.298
TG	0.734 (0.555-0.969)	**0.029**
APO-A	5.310 (0.883-31.941)	0.068
E2 on the trigger day	1.000 (1.000-1.000)	0.207
P on the trigger day	1.642 (0.915-2.945)	0.096
High quality embryo rate	1.025 (1.010-1.041)	**0.001**
High quality blastocyst rate	1.014 (1.002-1.026)	**0.025**

CLBR was treated as a binary outcome variable (live birth vs. non-live birth). Patients were followed for at least 12 months after the first embryo transfer. CLBR was defined as at least one live birth resulting from either fresh embryo transfer or subsequent frozen-thawed embryo transfers derived from the same controlled ovarian stimulation cycle. Non-live birth was set as the reference category in the multivariable logistic regression model. OR, odds ratio; CI, confidence interval.

Bold values indicate statistical significance (P < 0.05).

### Construction, validation, and performance evaluation of the prediction model

A nomogram for predicting CLBR was developed based on the eight variables retained from the previous selection process (**Figure 3**). Each predictor was assigned a corresponding score according to its regression coefficient. The total score, obtained by summing the individual scores, corresponds to a predicted probability ranging from 0.1 to 0.9.

**Figure 3:**
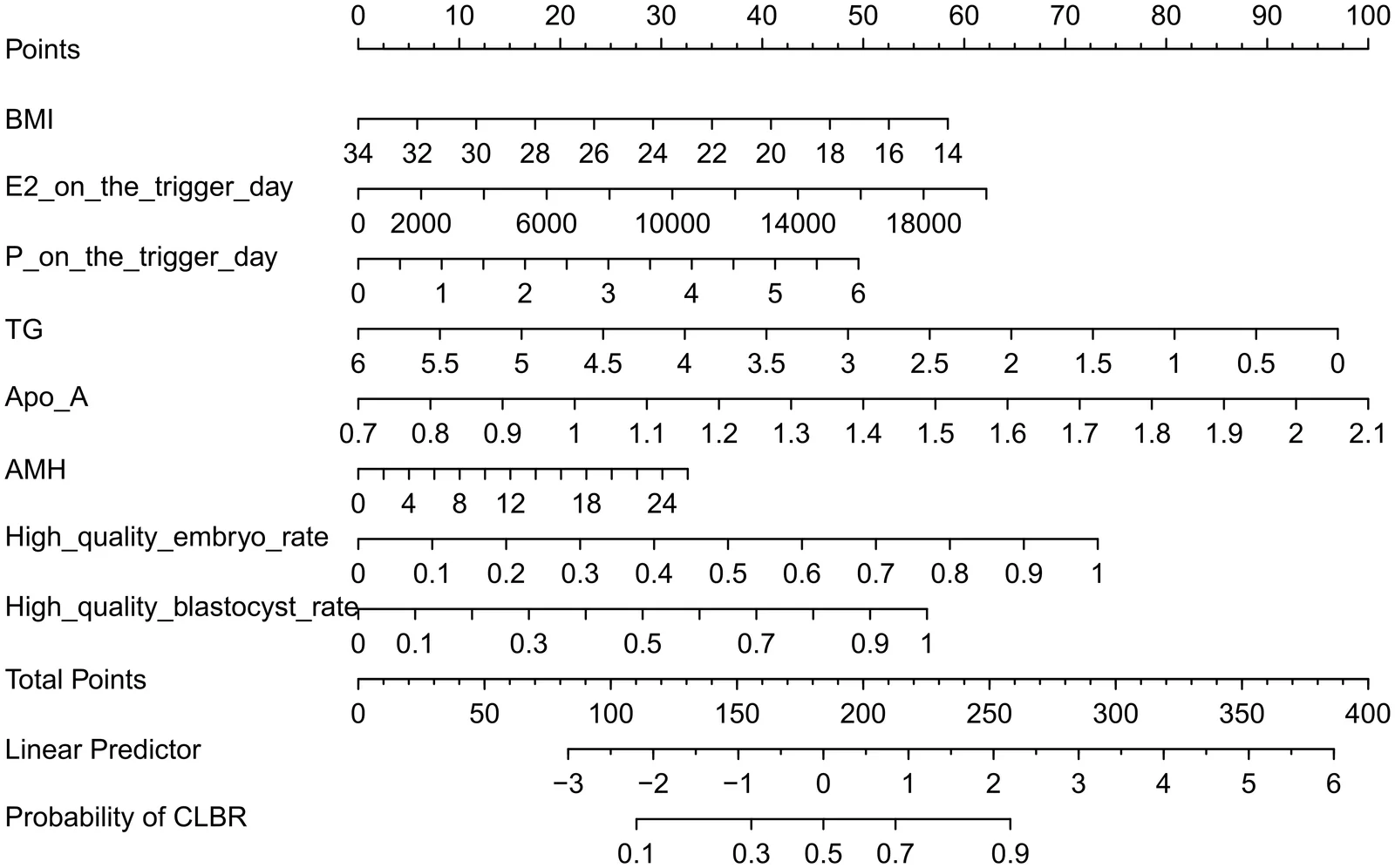
Nomogram for predicting CLBR after IVF/ICSI in PCOS patients.

The entire cohort was randomly divided into a training set (n = 290) and a validation set (n = 123) at a 7:3 ratio. Internal validation was performed using bootstrap resampling with 1,000 repetitions. The model achieved an AUC of 0.804 (95% CI: 0.745–0.862) in the training set and 0.760 (95% CI: 0.653–0.867) in the validation set (**Figure 4**).

**Figure 4:**
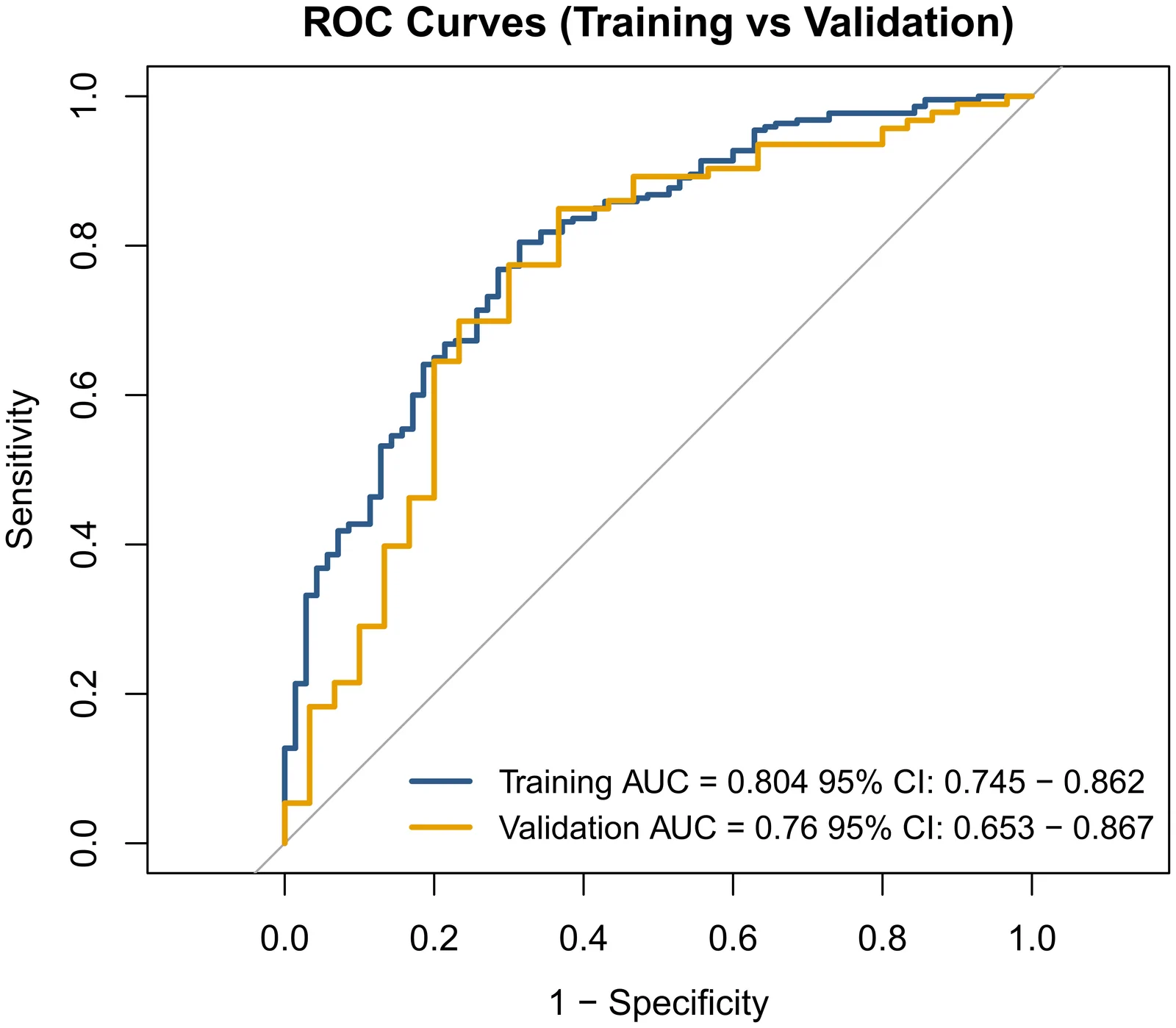
ROC curves for the training and validation sets.

Regarding discriminative ability, the model achieved an AUC of 0.804 in the training set and 0.760 in the validation set, with a bootstrap-corrected AUC of 0.775. For calibration, the Hosmer-Lemeshow test yielded *P* values of 0.728 and 0.091 for the training and validation sets, respectively. The Brier scores were 0.140 and 0.152, respectively. Calibration plots showed that the bias-corrected curve for the training set aligned closely with the ideal reference line. In the validation set, the calibration intercept was 0.311 and the slope was 0.734 (**Figures 5A, B**).

**Figure 5:**
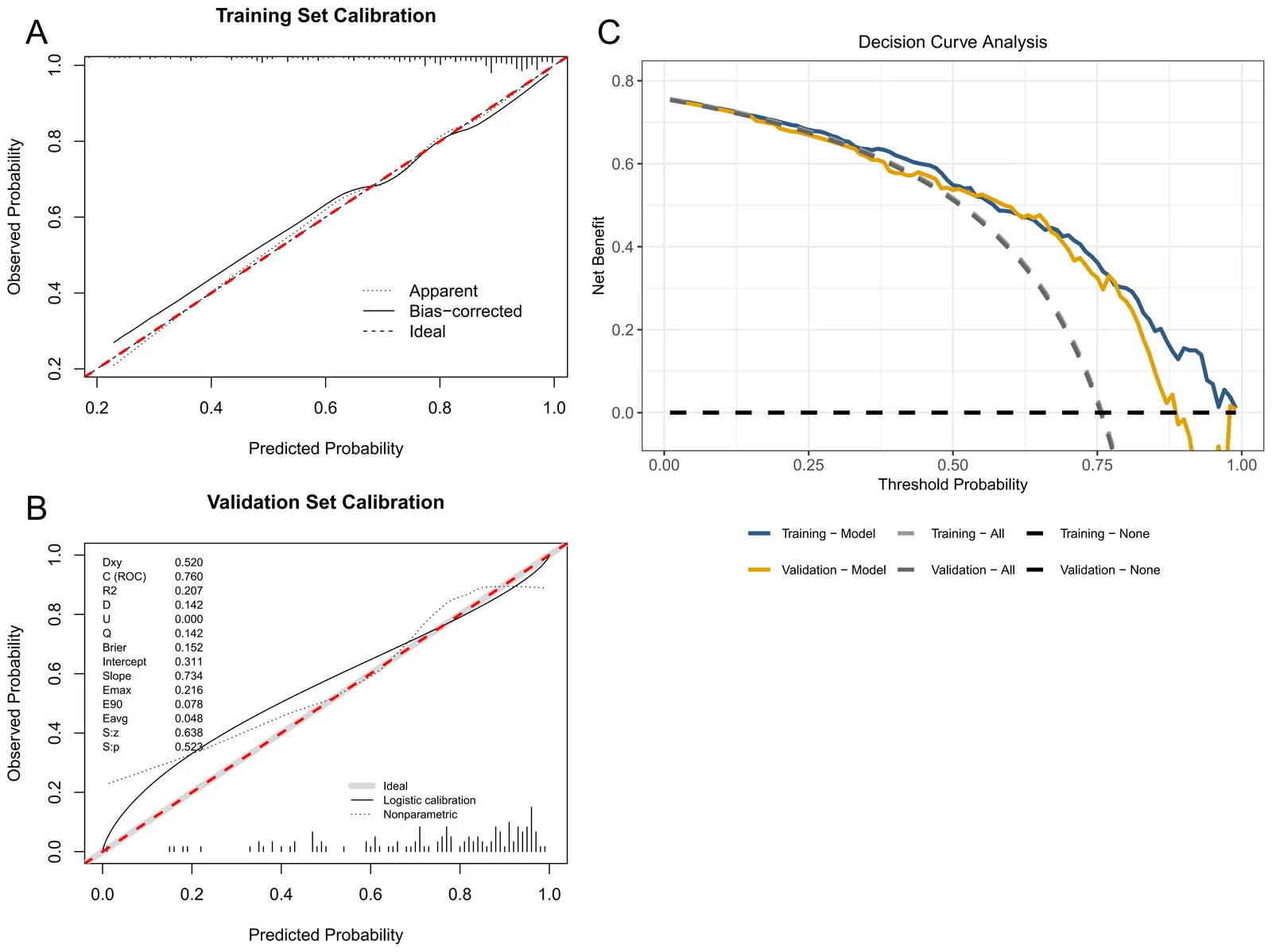
Calibration curves and decision curves analysis (DCA) of the prediction model. **(A)** Calibration curve for the training set. **(B)** Calibration curve for the validation set. **(C)** DCA curve for the training and validation set.

Decision curve analysis demonstrated that, across threshold probabilities ranging from 0.1 to 0.9, the model curves in both sets yielded higher net benefit than the “treat-none” strategy. In the training set, the model’s net benefit exceeded that of the “treat-all” strategy within the 0.2–0.9 range; in the validation set, this was observed within the 0.2–0.8 range (**Figure 5C**).

## Discussion

PCOS is a common endocrine and metabolic disorder characterized by ovulatory dysfunction, hyperandrogenism, and polycystic ovarian morphology. Women with PCOS frequently exhibit various metabolic abnormalities, with dyslipidemia being particularly prevalent (23). While much of the previous research has concentrated on the role of obesity and IR in ART outcomes, the specific contribution of dyslipidemia has not been fully elucidated. To address this gap, we carried out a retrospective analysis of 413 infertile PCOS patients undergoing their first IVF/ICSI cycle to systematically evaluate the association between lipid metabolic parameters and CLBR, and further developed a nomogram for individualized risk prediction.

### Dyslipidemia and ovarian response

PCOS patients with dyslipidemia needed significantly higher total Gn doses than those with normal lipid profiles, even after BMI adjustment, consistent with previous reports (9, 10, 24). One possible explanation is that dyslipidemia disrupts the hypothalamic-pituitary-ovarian axis, reducing the ovarian sensitivity to Gn (25). Correspondingly, among PCOS patients with dyslipidemia, we observed markedly lower E2 levels on the day of trigger, as well as fewer retrieved oocytes. The potential mechanisms underlying these observations include: hyperlipidemia can reduce serum sex hormone-binding globulin (SHBG) levels, leading to an increase in free testosterone, which in turn directly impairs ovarian response to Gn (25); additionally, inflammatory factors derived from adipose tissue, such as tumor necrosis factor-alpha(TNF-α), may inhibit oocyte maturation, promote apoptosis, and reduce the sensitivity of granulosa cells to Gn (26).

### Dyslipidemia and embryo quality

Multiple studies have confirmed that dyslipidemia adversely affects oocyte and embryo quality (11, 27, 28). A study on *in vitro* maturation in PCOS women indicated that dyslipidemia negatively impacted pregnancy outcomes in ART regardless of whether COH was performed (12). Hyperlipidemia generates excessive reactive oxygen species, inducing oxidative stress that damages granulosa cell endoplasmic reticulum, impeding oocyte maturation and blastocyst formation (29–31)., and causes mitochondrial impairment and meiotic errors, compromising oocyte quality and fertilization rates, and inducing embryonic defects (32, 33). In line with these mechanisms, our dyslipidemia group exhibited significantly lower numbers of high-quality embryos, blastocysts, and frozen embryos, as well as reduced first-cycle implantation rate and CLBR. Studies indicate that the adverse impact of dyslipidemia on ART outcomes is independent of obesity and PCOS status (34). In non-PCOS populations, the pregnancy rate in the normal lipid group was significantly higher than in the abnormal group (82.7% vs 74.4%, *P<* 0.001) (14), and TG levels were negatively correlated with live birth rate (35). Additionally, elevated TC and LDL-C are associated with lower live birth rates (36, 37), with elevated LDL-C also linked to lower pregnancy rates and higher miscarriage rates (37). Conversely higher HDL-C is correlated with more favorable outcomes (37). However, research on lipid metabolism in PCOS populations remains limited. Gao et al. found that high TC levels are associated with adverse pregnancy outcomes (15), and Xue Jiang et al. reported that TC level is negatively correlated with CLBR in PCOS patients (38). Our study further identified elevated TG as an independent risk factor for reduced CLBR, potentially through oxidative stress, endoplasmic reticulum stress, and lipotoxicity (30, 39, 40).

### Metabolic dysfunction as a driver of reduced CLBR

Patients with PCOS have a prevalence of dyslipidemia, with an incidence approximately 16.1% higher than that in the general population (41). Notably, this lipid metabolism disorder is also common in non-obese PCOS patients, exacerbating IR and other metabolic abnormalities, and significantly negatively affecting fertility (42). Pugh et al. reported that women with dyslipidemia have a 19–36% lower probability of conceiving naturally per cycle (43). Consistent with these findings, dyslipidemia in non-obese PCOS patients impairs clinical pregnancy outcomes (10). In our multivariable analysis, TG was independently associated with CLBR (OR = 0.734, *P* = 0.029). Yet elevated TG seldom occurs in isolation—insulin resistance, a common feature of PCOS, drives lipolysis, free fatty acid release, and hepatic VLDL production, ultimately leading to hypertriglyceridemia (44–46). Thus, raised TG may serve as a clinical marker of broader metabolic dysfunction rather than a direct toxic factor. Its relationship with CLBR likely reflects a combination of insulin resistance, chronic inflammation, and oxidative stress.

Our study showed that the live-birth group exhibited lower HOMA-IR and TG levels, alongside higher HDL and APO-A, suggesting that insulin resistance and lipid abnormalities may jointly contribute to adverse pregnancy outcomes in PCOS. Although HOMA-IR differed significantly between groups in baseline comparison, it was not retained in the Lasso regression, likely because its effect on CLBR was largely mediated by closely related metabolic factors such as BMI, TG, and HDL. Recently, Feng et al. (47)demonstrated that the triglyceride-glucose index was significantly negatively associated with live birth rate (OR = 2.32, 95% CI: 1.42–3.78, *P*< 0.01) in 956 PCOS patients, and was significantly correlated with insulin resistance and metabolic syndrome. These findings suggest that combined metabolic indicators incorporating TG may have superior risk stratification value over a single lipid parameter, corroborating our conclusion that TG independently affects CLBR.

Subgroup analysis revealed that among overweight/obese patients, those with dyslipidemia had a significantly lower clinical pregnancy rate than those with normal lipids (47.5% vs. 61.9%, *P* = 0.043). No such difference was seen in the normal-BMI group, suggesting a synergistic effect between obesity and dyslipidemia. Potential mechanisms include: (i) chronic inflammation impairing folliculogenesis (48, 49), and the increase of blood lipids especially triglyceride was positively correlated with TNF-a (10). (ii) an “obesity–IR–dyslipidemia” vicious cycle (35); and (iii) adipokine imbalance affecting oocyte quality and endometrial receptivity (26, 50). He et al. (51)also found that PCOS patients with high BMI had fewer oocytes and good-quality embryos, lower CLBR, and elevated IL-6 levels correlated with reduced live birth, suggesting BMI may affect ART outcomes via inflammatory responses. BMI was also an independent predictor of CLBR (OR = 0.915, *P* = 0.049), with each 1 kg/m² increase reducing live birth probability by ~8.5%. Overall, weight and lipid management should be prioritized in pre-conception care for PCOS patients, especially those with overweight/obesity and dyslipidemia.

### Dyslipidemia, endometrial receptivity, and implantation

Apart from affecting ovarian response and embryo quality, dyslipidemia in PCOS patients may also impair embryo implantation by damaging endometrial receptivity, leading to decreased embryo implantation rates and increased risk of early miscarriage (10, 52, 53)). In this study, the first-cycle implantation rate was significantly lower in the PCOS dyslipidemia group (44.0% vs. 55.7%, *P* = 0.009).The first-cycle clinical pregnancy rate and overall clinical pregnancy rate were also noticeably lower, though the differences were not statistically significant (57.4% vs. 66.1%, *P* = 0.084; 56.7% vs. 63.4%, *P* = 0.110). Further BMI-stratified analysis revealed that within the overweight/obese subgroup, the clinical pregnancy rate was significantly lower in those with dyslipidemia (47.5% vs. 61.9%, *P* = 0.043), supporting the aforementioned viewpoint. Although the first-transfer clinical pregnancy rate was lower in the dyslipidemia group, the difference was not statistically significant. A possible explanation is that during the first-transfer cycle, clinicians tend to prioritize the transfer of morphologically selected “optimal” embryos. This practice may partially offset the overall disadvantage in embryo quality associated with dyslipidemia, thereby maintaining a comparable clinical pregnancy rate. However, no significant difference in miscarriage rate was observed in this study. This negative finding may be attributable to the retrospective nature of our study, the limited sample size, and the multifactorial etiology of miscarriage.

### Age in PCOS populations: a less dominant factor

Similarly, age did not differ significantly between the live-birth and non-live-birth groups (*P* > 0.05). Several factors may explain this. First, only 10.4% of patients in our cohort were over 35 years old, and this restricted age range likely diminished the biological impact of age. Second, PCOS patients generally maintain a favorable ovarian reserve, with relatively high follicle counts and ovarian responsiveness even at advanced ages, which may partially offset age-related fertility decline. Third, the effect of age on pregnancy outcomes in PCOS may be masked or mediated by metabolic factors such as BMI, insulin resistance, and dyslipidemia. This observation aligns with previous reports suggesting that in PCOS populations, metabolic abnormalities may outweigh age as determinants of reproductive success (54).

### Nomogram performance and potential clinical value

We developed and internally validated a nomogram for predicting CLBR in PCOS patients undergoing their first IVF/ICSI cycle. The model integrated eight readily available clinical parameters: BMI, TG, APO-A, trigger-day E_2_ and P, high-quality embryo rate and high-quality blastocyst rate. The model achieved AUCs of 0.804 in the training set and 0.760 in the validation set, with a bootstrap-corrected AUC of 0.775, indicating moderate discriminative ability. Calibration was satisfactory, as reflected by a Brier score of 0.152 in the validation set and calibration curves showing good agreement between predicted and observed probabilities. Decision curve analysis confirmed a positive net benefit across clinically relevant thresholds.

In terms of discrimination, the model achieved AUCs of 0.804 and 0.760 in the training and validation sets, respectively, with a bootstrap-corrected AUC of 0.775, indicating moderate to good discriminative ability. The AUC declined by approximately 0.044 from the training set to the validation set, which is within an acceptable range and suggests that overfitting may not be a major concern. Furthermore, the bootstrap-corrected AUC (0.775) differed from the training set AUC (0.804) by only 0.029, further supporting acceptable generalizability of the model.

Regarding calibration, the validation set Brier score was 0.152 (< 0.25), and the calibration slope was 0.734, slightly below the ideal value of 1, suggesting some degree of probability bias in new data. This bias may be attributable to sampling variation due to the limited validation sample size (n = 123), or inherent calibration drift in independent samples. The calibration curves demonstrated generally acceptable agreement between predicted and observed probabilities, although slight deviations were observed in certain probability ranges. Decision curve analysis demonstrated that, across clinically relevant threshold ranges (0.2–0.8), the model’s net benefit exceeded that of the “treat-none” strategy, suggesting some reference value for clinical decision-making.

Of note, both high-quality embryo rate and high-quality blastocyst rate were retained in the final model. These two variables reflect embryo quality at different developmental stages—day 3 and days 5–6, respectively. Their simultaneous retention by Lasso regression suggests complementary rather than overlapping contributions to predicting CLBR.

This model may serve as a quantitative adjunct to clinical judgment in individualized prognostic counseling for PCOS patients undergoing IVF/ICSI. Prior to ovarian stimulation, BMI and TG can be used as preliminary risk stratification tools to guide lifestyle or pharmacological interventions. After embryo development, all eight variables can be integrated to provide a refined prediction of CLBR, thereby offering quantitative support for embryo transfer decisions and patient counseling. High-risk patients identified by the model (e.g., those with elevated TG or BMI) may benefit from more aggressive metabolic management, though this hypothesis requires prospective confirmation.

Nevertheless, We acknowledge that our model is not yet ready for direct clinical implementation. Its primary value at this stage lies in serving as a foundation for future multicenter prospective validation and in exploring the potential role of lipid parameters in IVF/ICSI prognosis. External validation in independent, larger corhots is urgently needed before any clinical application can be considered.

### Limitations

Several limitations should be acknowledged. First, this was a single-center retrospective study with a relatively small validation set (n = 123), which may have introduced instability into the performance estimates; larger cohorts are needed for further validation. Second, only internal validation (bootstrap) was performed, and external validation in independent multicenter cohorts is lacking. Third, some predictors (e.g., embryo quality rates) are only available after embryo development, limiting pre-treatment decision-making. Fourth, only baseline lipid levels were measured; dynamic changes during stimulation were not assessed. Fifth, potential confounders such as diet, exercise, and psychological status were not included. Sixth, detailed data on lipid-lowering or weight-loss interventions were not collected, precluding assessment of their impact on FET outcomes.

Future prospective, multicenter studies are required to externally validate the model and assess its generalizability. Furthermore, prospective studies with systematic collection of detailed data on lipid-lowering and weight-loss interventions are warranted to further evaluate their impact on FET outcomes in PCOS patients.

## Data Availability

The data analyzed in this study is subject to the following licenses/restrictions: The data presented in this study are available on request from the corresponding author due to ethical and privacy restrictions. Data are not publicly available because they contain information that could compromise the privacy of research participants. Requests to access these datasets should be directed to Huiling Xu, E-mail: xhlice@foxmail.com.
