# Supplementary material for: Associations of lipid metabolic parameters with cumulative live birth rate in PCOS patients undergoing IVF/ICSI and development of a nomogram-based predictive model
Source: Front Endocrinol (Lausanne). 2026 Sep 25;17:1924206. doi: 10.3389/fendo.2026.1924206 (PMC13649422; doi:10.3389/fendo.2026.1924206)
Supplement: Supplementary file 1 [file DataSheet1.pdf]

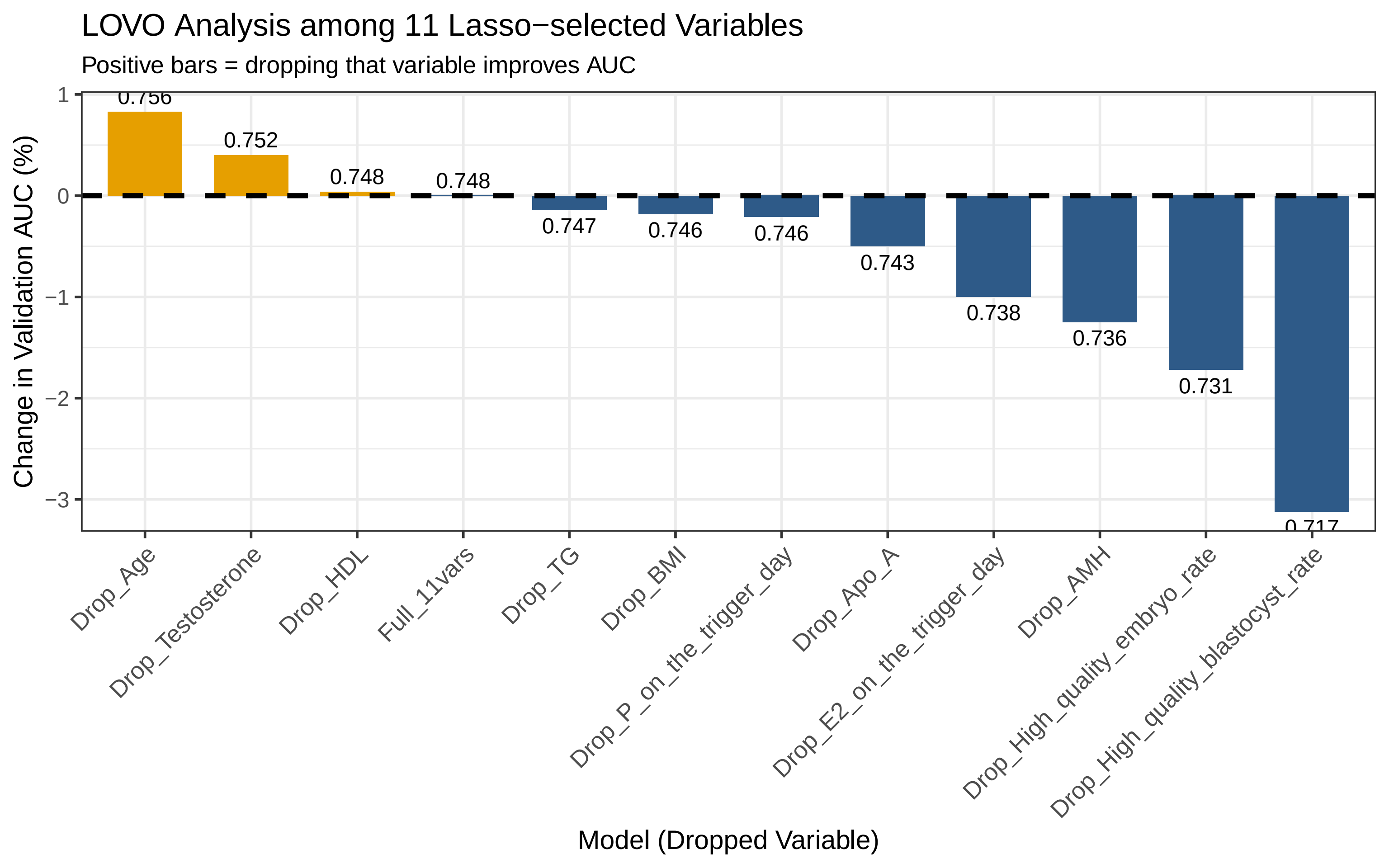
LOVO Analysis among 11 Lasso−selected Variables
Positive bars = dropping that variable improves AUC
Change in Validation AUC (%)
1
0
−1
−2
−3
0.756
0.752
0.748
0.748
0.747
0.746
0.746
0.743
0.738
0.736
0.731
0.717
Drop_Age
Drop_Testosterone
Drop_HDL
Full_11vars
Drop_TG
Drop_BMI
Drop_P_on_the_trigger_day
Drop_Apo_A
Drop_E2_on_the_trigger_day
Drop_AMH
Drop_High_quality_embryo_rate
Drop_High_quality_blastocyst_rate
Model (Dropped Variable)
